# Mortality in ICU COVID-19 Patients Is Associated with Neutrophil-to-Lymphocyte Ratio (NLR): Utility of NLR as a Promising Immunohematological Marker

**DOI:** 10.1155/2023/9048749

**Published:** 2023-11-09

**Authors:** Shahram Seyfi, Abbas Azadmehr, Khadijeh Ezoji, Majid Nabipour, Arefeh Babazadeh, Kiarash Saleki, Mehdi Mahmoodi, Amir Hossein Pouladi

**Affiliations:** ^1^Department of Anesthesiology, Babol University of Medical Sciences, Babol, Iran; ^2^Cellular and Molecular Biology Research Center, Health Research Institute, Babol University of Medical Sciences, Babol, Iran; ^3^Social Determinants of Health Research Center, Health Research Institute, Babol University of Medical Sciences, Babol, Iran; ^4^Department of Internal Medicine, Babol University of Medical Sciences, Babol, Iran; ^5^Department of Infectious Disease, Infectious Diseases and Tropical Medicine Research Center, Health Research Institute, Babol University of Medical Sciences, Babol, Iran; ^6^Student Research Committee, Babol University of Medical Sciences, Babol, Iran; ^7^Department of e-Learning, Virtual School of Medical Education and Management, Shahid Beheshti University of Medical Sciences (SBMU), Tehran, Iran; ^8^USERN Office, Babol University of Medical Sciences, Babol, Iran

## Abstract

**Background:**

Achieving a suitable medical laboratory index is very important for the prediction of clinical outcome of COVID-19 patients hospitalized to the intensive care unit (ICU). The correlation between neutrophil-to-lymphocyte ratio (NLR) and unfavorable outcome of COVID-19 patients hospitalized to ICU was the aim of this study.

**Methods:**

We evaluated a cross-sectional study of 312 COVID-19 patients who were hospitalized to the ICU (confirmed by PCR and CT-Scan), in Babol city, Mazandaran province. WBC, RBC, lymphocyte, neutrophil, monocyte, platelet count, NLR, C-reactive protein (CRP), ESR, MCV, MHC, and other factors were evaluated.

**Results:**

Our findings indicated that all patients aged 56 to 69 years with COVID-19 had a significant difference (*P* < 0.05) in neu, lymph, PLT count, NLR, ESR, Hb, and CRP. Also, NLR was significantly (*P* < 0.05) correlated with the death or discharge of the ICU hospitalized patients. The cut-off of NLR was 7.02 and the mean of NLR was 11.3 ± 10.93 and 5.8 ± 7.45 in death and discharge COVID-19 patients hospitalized to ICU, respectively. ROC curve indicated that, for NLR, the area under curve was 0.76.

**Conclusions:**

Our findings showed that NLR can be utilized as a clinical laboratory predictive parameter for mortality of COVID-19 patients admitted to ICU.

## 1. Introduction

COVID-19 was initiated by SARS-COV-2 and resulted in a worldwide public health emergency. This new virus has a single-stranded RNA genome and was first originated in Wuhan City, Hubei Province, China. Then, it spread rapidly from its source to other parts of the world [1–3]. To date, there is no specific and fully effective vaccine or antiviral drug to control and eradicate this problematic virus. Also, previous studies have shown that natural products exert versatile therapeutic effects including immunomodulatory antitumor, anti-inflammatory roles. In fact, current evidence suggests that herbal medicine added to standard treatment has potential benefits in the treatment of COVID-19 symptoms [4–8].

While many patients have mild to moderate clinical symptoms, a number of patients experience severe uncontrolled inflammatory immune response reactions, that lead to acute lung damage, hypoxemia, and ultimately respiratory failure. Acute respiratory distress syndrome (ARDS) is the major cause of mortality in patients with COVID-19 [9].

In addition, the laboratory parameters especially cell blood count (CBC) have been found to be useful for distinguishing severe and nonsevere cases. The distinction between high-risk and low-risk infected individuals in the intensive care unit (ICU) allows us to optimize the allocation of limited resources in these situations. A discerning pattern of abnormalities of blood parameters and inflammatory factors can be observed among patients with or without severe or critical illness [10].

Recently, a study showed that “Cytokine storm” as a consequence of the extensive proinflammatory response activated by coronavirus infection could potentially damage local and systematic tissues and reduce lymphocyte count [11]. Other studies have extended the inflammatory markers beyond usual cytokines, chemokines, and usual laboratory tests. We recently suggested soluble Fas ligand (sFasL) as a prognostic marker for mortality and severity of COVID-19. Moreover, we indicated that sFasL could be an inflammatory marker that mediates the cytokine storm in COVID-19 via affecting inflammatory cells, such as neutrophils and lymphocytes [12, 13].

On the other hand, immunohematological parameters including lymphocyte, neutrophil platelet count, and neutrophil-to-lymphocyte ratio (NLR) are simple and accessible parameters that are related to COVID-19 mortality and severity in patients admitted to the ICU. Results from several studies have shown that the immunohematological laboratory parameters as prognostic markers are useful for the identification of patients with high-risk and low-risk in the ICU and also for therapeutic management of COVID-19 [14–18]. The identification and use of laboratory and clinical parameters as prognostic factors are essential for the management of severe forms of this disease. In the present work, we evaluated the association between NLR and unfavorable outcomes (fatal or nonfatal) of COVID-19 patients hospitalized to ICU.

## 2. Materials and Methods

This research followed a cross-sectional design, comprising COVID-19 patients visited in Ayatollah Rouhani hospital in Babol city, Mazandaran province, and hospitalized to the ICU between 2020 and 2021.

In this study, the statistical sample included 312 ICU hospitalized COVID-19 patients (aged 56 to 69 years) which confirmed by PCR and CT Scan. Our study protocol was approved by the Ethical Committee of Babol University of Medical Sciences (IR.MUBABOL.REC.1399.498). The samples were divided into two groups including improved or died. Disease outcomes were registered.

CBC difference parameters including WBC, RBC, lymphocyte, neutrophil, monocyte, and platelet count, Hb, HCT, MCV, MCH, MCHC, RDW-SD, RDW-CV, and PDW were measured by hematology auto-analyzer (HITACHI-Roche 902, JP and Sysmex KX-21N, JP). Also, ESR, CRP, and NLR were evaluated. Data were analyzed using the Mann–Whitney nonparametric test, logistic regression model, and receiver operating characteristic (ROC) curve.

To investigate the relationship between independent variables and the occurrence of the final event including death or survival of COVID-19 patients, after examining the normality using the Kolmogorov–Smirnov test, the Mann–Whitney nonparametric test was used. In order to predict the possibility of a COVID-19 patient's death based on blood cell counts and inflammation markers, a logistic regression model was fitted to the data. Also, independent predictor variables were entered into the model using the enter input method. Finally, analyses for available data were performed using Statistical Package for Social Sciences (SPSS) 26 and Prism Version 8.4.3 software at a significance level of 0.05.

## 3. Results

In this study, 312 patients with severe COVID-19 hospitalized in the ICU at Ayatollah Rouhani Hospital in Babol were included. Our findings indicated that 158 patients (51.6%) died and 154 Patients (49.4%) were discharged from the hospital. Descriptive information and mean differences for laboratory parameters of the CBC test, including blood cells and markers of inflammation in hospitalized patients, based on the final event of death or discharge are presented in Table 1.

WBC, neutrophils and lymphocytes, RBC, platelets count, hemoglobin (Hb), and hematocrit were significantly different when comparing hospitalized patients who died to those who were discharged (*p* < 0.05). However, laboratory parameters such as MCV and MCH were not significantly different between the studied groups.

Moreover, there was a significant difference for CRP (*p* < 0.0001) and ESR (*p* = 0.018), which are two biomarkers of inflammation, between the two groups of patients who died and were discharged.

In order to predict survival of COVID-19 patients based on blood cell counts and parameters of inflammation in patients, the logistic regression model was performed and the results are presented in Table 2. Independent predictor variables were entered into the model using the enter input method.

According to the results of logistic regression model fitting (Table 2), parameters related to neu, lym, RBC, CRP, ESR, and NLR could meaningfully predict the possibility of final mortality in patients with severe COVID-19, but other laboratory parameters related to blood cells were not significant predictors for death.

Based on odds ratio (OR) analysis, the percentage of neutrophils (OR = 1.096) and lymphocytes (OR = 0.930), it can be said that, for one unit of increase in the neutrophil index (*β* = 0.091) and lymphocytes (*β* = −0.071) of the COVID-19 patient, the probability of occurrence of death increases by 9.1% and decreases by 7.1%, respectively.

In addition, our findings indicated that an increase in the RBC count (OR = 0.515, *β* = −0.662) was a predictor for a decreased probability of death in COVID-19 patients.

**Figure 1:**
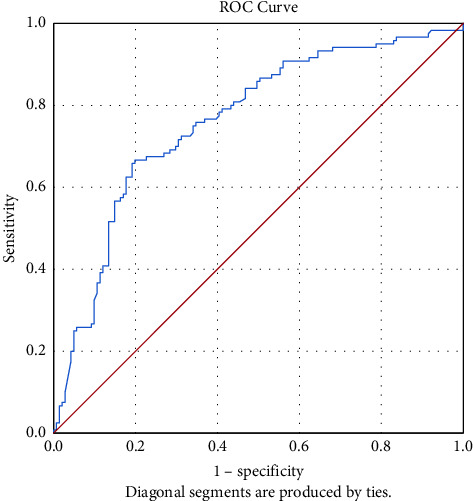
ROC curve for predicting the cut-off value of NLR for death outcome. Area under the curve was 0.76.

Moreover, the optimal cut-off for NLR was 7.02. Mean NLR values were 11.3 ± 10.93 and 5.8 ± 7.45 in death and discharge of COVID-19 patients hospitalized to ICU, respectively. The cut-off point as a prognostic factor of death in severe COVID-19 patients for NLR was 7.02 with 63% sensitivity and 83% specificity (Figure 1). The area under the curve (AUC) for the NLR ROC curve was 0.76.

In this study, 43% and 46% of males and females expired, respectively. Gender was not significantly different in dead cases compared to alive cases (*p* > 0.05). Moreover, we analyzed NLR results for male and female subpopulations. 61.92% of cases were male and 38.08% were female. Additionally, ROC analyses showed optimal cut-off points for the outcome death were 6.12 and 4.59 in males and females, respectively. Regression analysis of NLR as a predictor of death in males (*p* = 0.0002) and females (*p* = 0.02) showed that increased NLR predicted a higher risk of death in COVID-19 patients.

## 4. Discussion

The identification of available and inexpensive laboratory and clinical parameters as a fast prognostic marker is important for preventing progression of patients into the fatal form of COVID-19. Here, we investigated hematological laboratory parameters and association between NLR and unfavorable outcome (fatal or nonfatal) in COVID-19 patients hospitalized to ICU.

The first immune cells to reach the infected and inflammatory site in tissues are neutrophils. Also, it has shown that excessive lung hyperinflammation and damage in COVID-19 patients are associated with neutrophil uncontrolled hyperactivity [19].

Our findings showed that lymphocyte counts were lower while neutrophil counts were higher in dead compared to discharged cases with COVID-19. In addition, platelet counts were significantly lower between death and discharge groups in COVID-19 patients. Significant lymphopenia, thrombocytopenia, and conversely neutrophilia were found in COVID-19 patients in our study.

Similar to previous studies, our results indicated that the optimal NLR cut-off value was 7.02 and the mean of NLR in the COVID-19 patient's death groups (11.3 ± 10.93) was about two times higher compared to discharge groups (5.8 ± 7.45) from ICU [20].

The NLR as a laboratory parameter is an inexpensive and rapidly available hematological assay. Previous studies suggested that the NLR can be effectively used as an indicator of severity inflammation in COVID-19 patients [21–24]. A recent study demonstrated that the NLR cut-off value was from 1 to 13.39 for the severity categorizing of COVID-19 patients [21]. Also, an up-to-date systematic review and meta-analysis concluded that, on admission, NLR predicts both severity and mortality in COVID-19 cases, and an NLR value higher than 6.5 was related to a highly increased mortality risk, which was similar to the cut-off value suggested by our study [25]. Another study indicated a suitable value for NLR as a predictive marker for COVID-19 patients complicated with cardiovascular disorders. Results of their study showed that follow-up NLR had better overall predictive value, compared to baseline NLR, indicating the importance of time of sample collection for the prediction of mortality in complicated COVID-19 patients [26]. Also, in this regard, a multi-inflammatory index along with CRP and NLR has been studied recently [27, 28].

We investigated the accuracy of the NLR parameter in forecasting the severity of COVID-19 patients admitted to ICU. In predicting the possibility of final death of COVID-19 patients, some laboratory parameters including neutrophil, lymphocyte percentage, and NLR may be a risk factor, while the two laboratory parameters mean volume and number of red blood cells may be a protective factor. For the future, we suggest design of therapeutics targeting inflammatory pathways in COVID-19. Using *in silico* vaccinology to target key inflammatory molecules may be a rapid and promising approach to achieve this goal [29, 30].

## 5. Conclusions

Briefly, our findings in this study indicated that the optimal cut-off for NLR as a mortality prognostic factor was 7.02, indicating that the COVID-19 patients hospitalized in ICU have NLR more than the cut-off were more likely to experience the high-risk outcome of the study which was death. So, NLR could be utilized as a clinical laboratory predictive parameter for the unfavorable outcome of COVID-19 patients hospitalized in ICU.

## Figures and Tables

**Table 1:** The difference between the mean of hematological parameters based on the outcome.

Parameter	Death (mean ± SD) (*n* = 158)	Discharge (mean ± SD) (*n* = 154)	*p* value
WBC (/*μ*L)	18981.64 ± 36343	12394.15 ± 40979	**<0.0001**
NEU (%)	84.01 ± 11.93	72.12 ± 12.48	**<0.0001**
LYM (%)	12.83 ± 12.18	19.92 ± 9.66	**<0.0001**
NLR	11.3 ± 10.93	5.8 ± 7.45	**<0.0001**
RBC (×10^6^/*μ*L)	3.99 ± 0.94	4.45 ± 0.72	**<0.0001**
Hb	10.97 ± 2.43	12.51 ± 2.14	**<0.0001**
HCT (%)	35.26 ± 28.43	36.95 ± 5.41	**<0.0001**
MCV (fl)	83.18 ± 9.5	88.25 ± 60.09	**0.545**
MCH	27.73 ± 4.02	28.29 ± 3.33	**0.074**
MCHC	32.87 ± 2.26	35.68 ± 24.75	**<0.0001**
RDW-SD	47.66 ± 8.8	42.45 ± 4.8	**<0.0001**
RDW-CV	15.5 ± 3.8	13.59 ± 1.91	**<0.0001**
PLT (/*μ*L)	183763.24 ± 132951.34	216201.29 ± 144726.8	**0.0004**
PDW (%)	13.46 ± 3.26	18.93 ± 71.59	**0.175**
CRP (mg ⁄L)	119.06 ± 88.23	77.49 ± 66.09	**<0.0001**
ESR (mm ⁄hour)	46.99 ± 32.68	56.27 ± 35.33	**0.018**

*p* values are provided in bold font, which are important as significance measures.

**Table 2:** Regression analysis of the chances of survival of patients with severe type COVID-19 based on hematological parameters.

Parameter	*β*	SE	OR	95% CI	*p* value
WBC (×10^3^/*μ*L)	4.996*e* − 006	3.534*e* − 006	1.000	1.000 to 1.000	**0.157**
NEU (%)	0.091	0.013	1.096	1.069 to 1.126	**<0.0001**
LYM (%)	−0.071	0.014	0.930	0.904 to 0.955	**<0.0001**
RBC (×10^6^/*μ*L)	−0.662	0.149	0.515	0.380 to 0.684	**<0.0001**
HCT (%)	−0.004	0.007	0.995	0.973 to 1.007	**0.507**
MCV (fl)	−0.005	0.008	0.994	0.970 to 1.002	**0.505**
PLT (×10^3^/*μ*L)	−1.889*e* − 006	9.667*e* − 007	1.000	1.000 to 1.000	**0.051**
CRP (mg ⁄L)	0.007	0.001	1.008	1.004 to 1.012	**<0.0001**
ESR (mm ⁄hour)	0.008	0.003	1.008	1.001 to 1.015	**0.021**
NLR	0.114	0.024	1.121	1.072 to 1.179	**<0.0001**

*P* < 0.05 was statistically significant. SE, standard error; OR, odds ratio; CI, confidence interval. Regression analyses are univariate simple regression. *p* values are provided in bold format, as it is an important significance measure.

## Data Availability

The data used in this study are available from the corresponding author on reasonable request.
